# Advanced glycation end products in diabetic retinopathy and phytochemical therapy

**DOI:** 10.3389/fnut.2022.1037186

**Published:** 2022-11-18

**Authors:** Qingzheng Kang, Haiyu Dai, Suwei Jiang, Li Yu

**Affiliations:** ^1^Department of Hematology and Oncology, International Cancer Center, Shenzhen Key Laboratory, Shenzhen University General Hospital, Shenzhen University Clinical Medical Academy, Shenzhen University Health Science Center, Shenzhen University, Shenzhen, China; ^2^School of Medicine, Shenzhen University, Shenzhen, China

**Keywords:** AGEs, diabetic retinopathy, RAGE, pathogenetic roles, antioxidant phytochemicals

## Abstract

Advanced glycation end products (AGEs) are generated by the nonenzymatic glycation of proteins or lipids. Diabetic retinopathy (DR) is one common complication in patients with diabetes. The accumulation of AGEs in retinal cells is strongly associated with the development of DR. AGEs can induce the breakdown of redox balance and then cause oxidative stress in retinal cells, exerting cytopathic effects in the progression of DR. The interaction between AGEs and the receptor for AGE (RAGE) is involved in multiple cellular pathological alterations in the retina. This review is to elucidate the pathogenetic roles of AGEs in the progression of DR, including metabolic abnormalities, lipid peroxidation, structural and functional alterations, and neurodegeneration. In addition, disorders associated with AGEs can be used as potential therapeutic targets to explore effective and safe treatments for DR. In this review, we have also introduced antioxidant phytochemicals as potential therapeutic strategies for the treatment of DR.

## Introduction

Advanced glycation end products (AGEs), a group of heterogeneous complexes, are produced from non-enzymatic glycation that happens between reducing sugars, a free amino group, nucleic acids, proteins, or lipids through the Maillard reaction (1). The formation of AGEs occurs either from foods or metabolized biological process in the physiological system. Foods containing high protein, sugar, fat, moisture, etc., easily result in the formation and accumulation of AGEs during processing or storage. Long-term AGE intake can lead to their cumulation in body fluids, contributing to the outbreak of chronic disorders and toxic pathogenesis in our body (2), especially diabetic complications. In addition, the generation of AGEs produces free radicals—reactive oxygen species (ROS) to facilitate redox imbalance, finally resulting in oxidative stress, and ROS can in turn prompt the formation of AGEs. ROS attacks functional biomacromolecules such as deoxyribonucleic acid (DNA), proteins, and lipids, subsequently affecting normal biological activity and inducing physiological dysfunction.

Diabetic retinopathy (DR) can impose a sight-threatening effect on the eyes, and it is generally classified as a microvasculature complication in diabetes (3). It is commonly recognized that DR is the main reason for vision loss or visual damage among elderly people or working-age adults worldwide (4). The amount of patients influenced by DR will ascend to 191 million in 2030 (5). Despite numerous studies, the mechanism underlying hyperglycemia-induced pathology in the retina still remains elusive. It has been shown that hyperglycemia-mediated progression of retinopathy is tightly associated with the abnormality of multiple metabolic pathways, in which the accumulation of AGEs plays a critical role (5). AGEs, such as carboxyethyllysine (CEL), carboxymethyllysine (CML), and pentosidine, have been well chemically characterized in the body. The occurrence of these AGEs is closely correlated with the severity of DR. For example, CML has been usually found in the retinal blood vessels of patients with diabetes (6). In addition, in the formation process of AGEs, proteins are structurally impaired by this non-enzymatic cross-link between amino groups and reducing sugars. One instance is the nonenzymatic glycation of elastin and collagen that are deeply involved in the formation of the stiffer blood vessels in DR (7).

## Formation mechanism of AGEs

**Figure 1:**
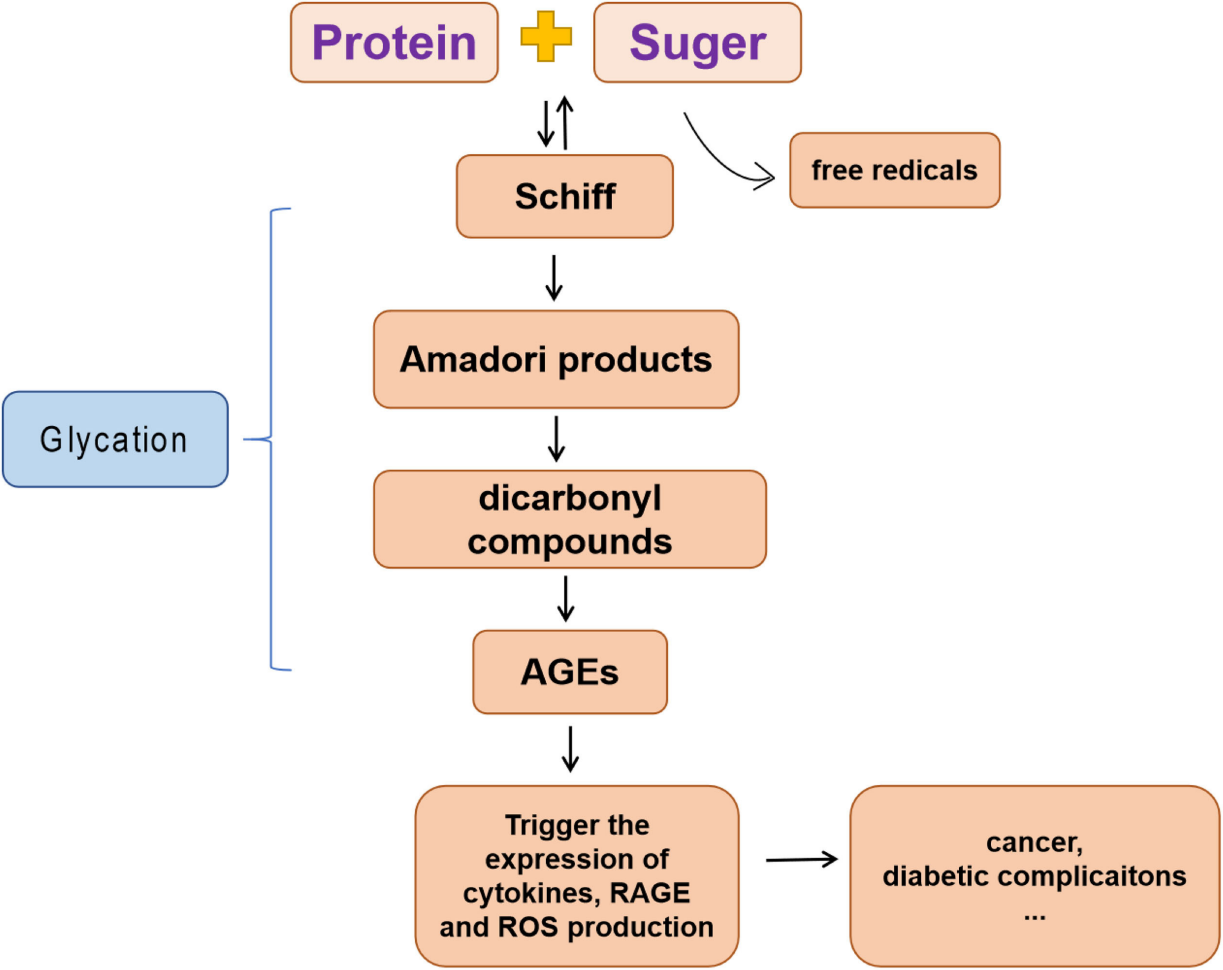
The formation of advanced glycation end products (AGEs). A schematic representation of the formation of AGE and the interaction with the receptor for AGE (RAGE). Glycation includes three main levels and promotes various diseases, such as diabetic complications, cancer, and so on.

The reaction between proteins and reducing sugars or carbonyl groups is known as the Maillard reaction, which non-enzymatically alters the function and morphology of these biological molecules (8). It mainly includes three levels (Figure 1). The primary level is the formation of the Schiff base in the reaction of free amino groups and glucose. In the existence of acid–base catalysis, the Schiff base is unstable and undergoes rearrangements to generate stable glycation—Amadori products that are early glycosylation products and mainly carbonyl compounds (Figure 1) (9). The second level produces reactive dicarbonyl compounds through chemical reactions of oxidation and dehydration, generating the precursors of AGEs (Figure 1). These reactive dicarbonyl compounds, such as glyoxal and deoxyglucosones compounds, react with free amino groups, proteins, DNA, or lipids (9) and undergo further cyclization reactions (the third level) to produce irreversible AGEs (Figure 1).

## AGEs and the receptor for AGE (RAGE) axis in DR

**Figure 2:**
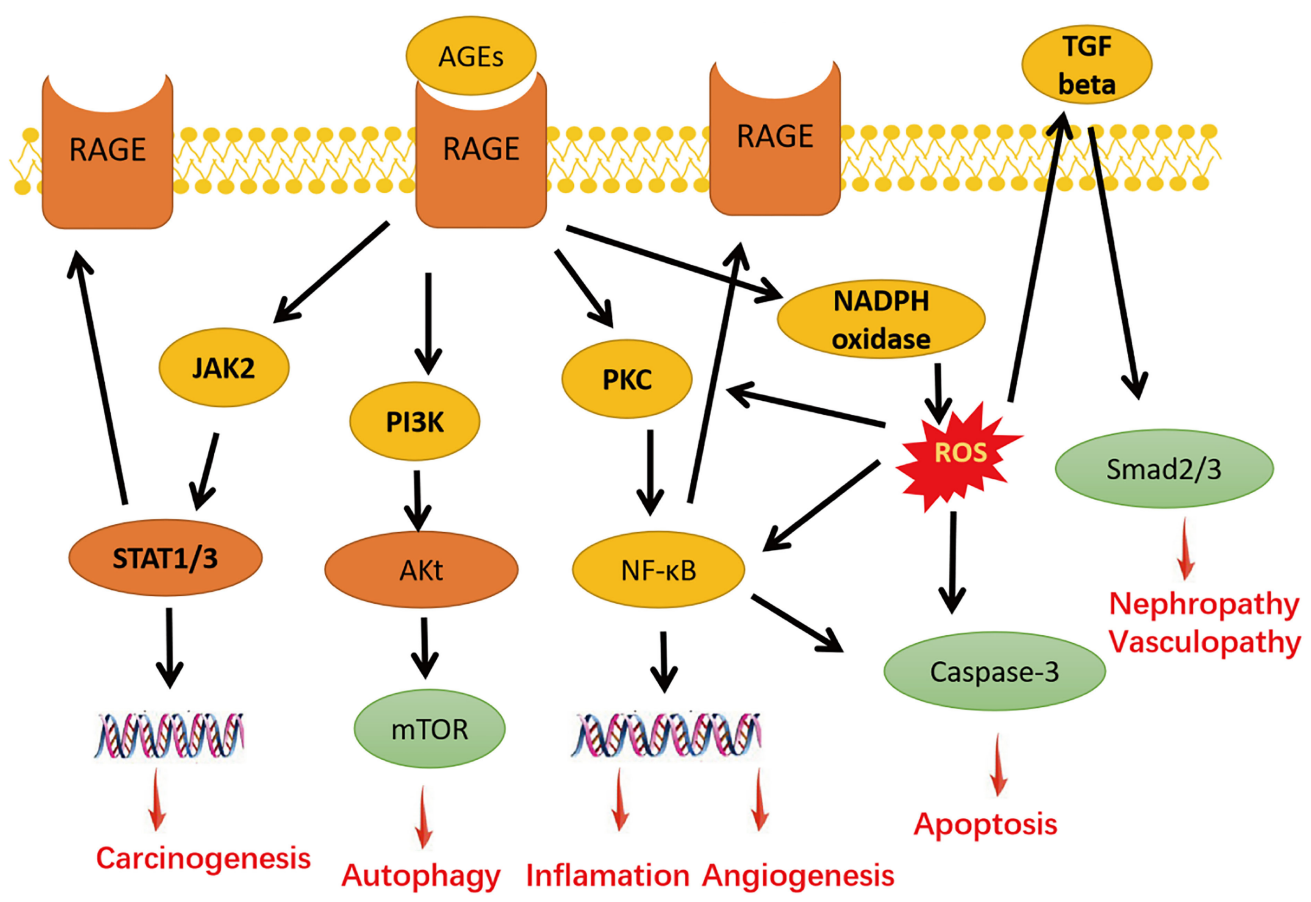
The AGE and RAGE axis and its downstream signaling pathways. The interaction of AGE–RAGE activates downstream signaling, including nuclear factor-κB (NF-κB) signaling pathway, transforming growth factor-β (TGF-β) pathway, Jak-STAT pathway, PI3K-Akt pathway, to promote inflammation, apoptosis, autophagy, angiogenesis, and nephropathy and vasculopathy, and induces the generation of reactive oxygen species (ROS) through nicotinamide adenine dinucleotide phosphate (NADPH) oxidase.

Advanced glycation end products and the receptor for AGE (RAGE) axis comprises AGE and RAGE. AGE-mediated damage occurs mainly through its interaction with RAGE on the cell membrane. The binding of AGEs and RAGE activates downstream signaling pathways, such as nuclear factor-κB (NF-κB) signaling pathway, transforming growth factor-β (TGF-β) pathway, Jak-STAT pathway, PI3K-Akt pathway, and so on, which are involved in the cellular processes of inflammation, apoptosis, autophagy, carcinogenesis, angiogenesis, and nephropathy and vasculopathy (10) (Figure 2). The binding of RAGE with AGEs can prompt the activation of the inflammatory factor NF-κB, which subsequently leads to pericyte apoptosis, and can also augment the expression of vascular endothelial growth factor (VEGF) to incur vascular endothelial permeability in the retina (6, 11). AGEs and the RAGE axis can also boost the expression of RAGE through enhanced downstream cellular signaling pathways (5).

The interaction of AGEs and RAGE generates ROS *via* activating nicotinamide adenine dinucleotide phosphate (NADPH) oxidase, which induces the activation of NF-κB and the elevation of proinflammatory cytokines. Proinflammatory cytokines upregulate the expression of NADPH oxidase (Nox) and augment the production of ROS (12). The production of AGE-induced ROS also participates and plays an important role in the pathophysiological progression of DR (13, 14). Periodic injection of AGEs into rats can cause retinal hyperpermeability and leukostasis while augmenting the level of RAGE and ROS; however, concurrent injection of pigment epithelium-derived factor (PEDF) can block this process *via* suppressing AGE-mediated ROS generation, NF-κB activity, and VEGF levels (15–17). Additionally, it has been suggested that the production of ROS can also be stimulated by AGEs *via* mitochondrial electron transport chain (18). As a consequence, the enrichment of ROS contributes to the accumulation of AGE (19) and the upregulation of RAGE (20), to aggravate all AGE-induced damages.

## Pathogenetic roles

### AGEs and metabolic abnormalities in DR

**Figure 3:**
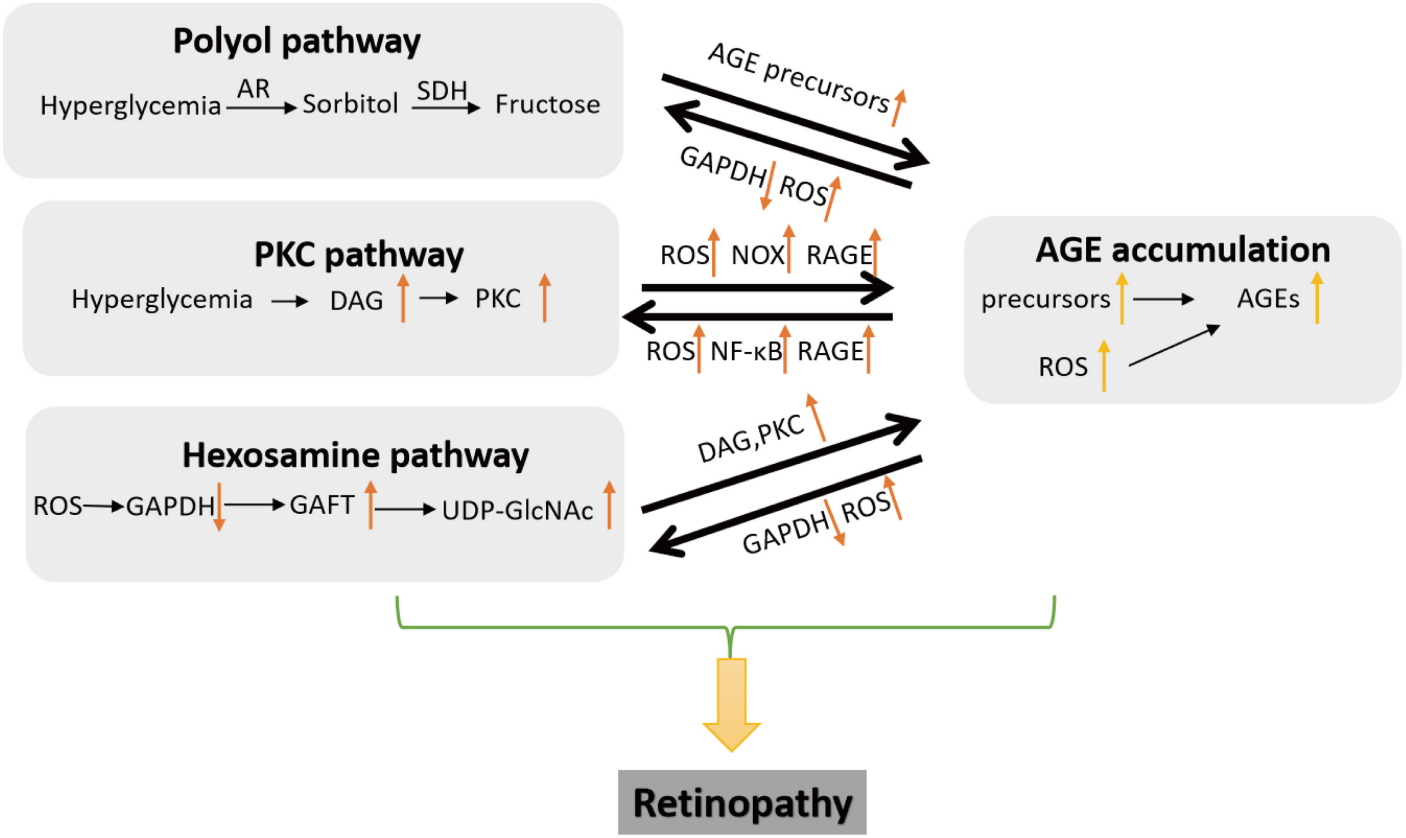
Metabolic abnormalities in a diabetic complication. Major disorders are polyol pathway, hexosamine biosynthesis pathway, and the activation of protein kinase C (PKC). Polyol pathway promotes the generation of AGE precursors. The activation of PKC induces the expression of vascular endothelial growth factor (VEGF) and RAGE and increase of ROS. Hexosamine pathway causes glycosylation of transcription factors and the generation of diphosphate uracil-N-acetylglucosamine (UDP-GlcNAc). These disorders interact with AGEs to promote the pathogenesis of diabetic retinopathy (DR).

Metabolic disorders play a critical role in the progression of DR, including enhanced glucose influx *via* hexosamine and polyol pathways and hyperactivated protein kinase C (PKC) pathway. These disorders promote the accumulation of AGEs in retinal cells and interact with AGEs to amplify these metabolic disorders, leading to the dysfunction of retinal tissues (Figure 3).

#### AGEs and polyol pathway

Glucose metabolism in polyol pathway is active in the hyperglycemic situation, which facilitates the formation of AGEs (21). In this pathway, glucose is catalyzed by aldose reductase, the first and rate-limiting enzyme, for its conversion into sorbitol with NADPH as the electron donor. Then, the sorbitol dehydrogenase oxidizes sorbitol into fructose, combined with the transfer of NAD into NADH (22, 23). In this process, the intermediate sorbitol, as hydrophilic alcohol, is difficult to diffuse through the cellular membrane, which brings about cell hypertonicity and the augmented osmotic pressure to finally induce osmotic damage of a retinal capillary (5). Notably, fructose produced as a byproduct can be phosphorylated into fructose-3-phosphate and then degenerated into 3-deoxyglucosone, both of which can be used as precursors to generate AGEs through non-enzymatic glycosylation (24).

#### AGEs and PKC pathway

Protein kinase C pathway is also deeply involved in the modulation of multiple physiological alterations in retinal tissues, including endothelial permeability, the upregulation of VEGF, retinal hemodynamics, and the adhesion and hyper-activation of leukocytes (leukostasis) in retinal cells (23, 25, 26). In addition, Nox can be positively modulated by PKC, which facilitates the generation of ROS in numerous vascular cells, such as pericytes, endothelial cells, and others (27, 28). The enrichment of ROS production in turn prompts the formation of AGE. Extracellular AGEs can combine its transmembrane receptor RAGE to activate PKC pathway to intensify this process (29).

#### AGEs and hexosamine pathway

Patients with diabetes have higher levels of hexosamine in their retinal cells. Glucose in hexosamine pathway is transferred into fructose-6-phosphate through phosphorylation. Subsequently, fructose-6-phosphate can be catalyzed into glucosamine-6-phosphate (5). Glucosamine-6-phosphate can be acetylated and isomerized to generate N-acetylglucosamine-6-phosphate and then transferred into the end products—diphosphate uracil-N-acetylglucosamine (UDP-GlcNAc). The end products can be utilized as substrates to produce glycosyl side chains for modifying lipids or proteins, which is called O-GlcNAc Modification—one particular type of glycosylation occurred in multiple chronic diseases (30, 31).

All metabolic disorders in DR interact with each other through the corresponding mid-components, AGEs or ROS (32). An elevated ROS level suppresses the activity of glyceraldehydes-3-phosphate dehydrogenase (GAPDH), which triggers the increased glucose influx into polyol pathway and increases intracellular AGEs (33). Meanwhile, the repression of GAPDH also leads to the activation of PKC and NF-κB, and the initiation of hexosamine pathway (34). Fructose-6-phosphate amidotransferase (GFAT) in hexosamine pathway is correlated with diacylglycerol- (DAG-) stimulated PKC activity (35). Excess glucose is metabolized in polyol pathway to form sorbitol and subsequently converted into fructose, which prompt the formation of strong glycosylating precursors for the generation of AGEs (36). Correspondingly, excessive AGEs interrupt redox balance, leading to oxidative stress and activating PKC pathway (37).

### AGEs and lipid peroxidation in DR

It has been discovered that tissues from DR contain a variety of metabolic components of lipid peroxidation (38), which are positively correlated to the duration and severity of diabetic complications (39). AGE-induced ROS generation brings about the breakdown of redox balance and prompts the rise of lipid peroxidation in DR. The abundance of polyunsaturated fatty acids (PUFAs) in retinal outer photoreceptor segment membranes makes the retina susceptible to ROS attack (40), and these PUFAs play an important structural and functional role in the retina. In the retina, arachidonic acid (AA), docosahexaenoic acid (DHA), and oleic acid (OA) are the main PUFAs, respectively, accounting for about 8, 50, and 10% in total fatty acids (41). Retinal lipid peroxidation not only jeopardizes photoreceptor membrane cells but also influences the normal physiological activity. The components in the process of lipid peroxidation, hydroxynonenal (HNE) and hydroxyhexenal (HHE), can chemically interact with cellular macromolecules (proteins or DNA), subsequently resulting in photoreceptor membrane damage and retinal pigment epithelial disorders (42). Additionally, ROS-induced mitochondrial dysfunction facilitates lipid accumulation in glia cells, which can be oxidized to render retinal neurodegeneration (43). As a result, one component in the production of AGEs is peroxidized lipids, and another method in the production of AGEs is by incubating lipid peroxidation products with proteins is (44).

### AGEs and structural and functional changes in DR

Advanced glycation end products-associated metabolic disorders give rise to various pathophysiological changes in the structures and functions of retinal microvasculature. Major alterations in DR include the thickened retinal capillary basement membrane (CBM), the breakdown of the blood–retinal barrier (BRB), and the formation of acellular and occluded capillaries.

One typical characteristic of DR is the thickening of the CBM, which is due to the disruption of the balance between the expression and degeneration of extracellular matrix (ECM) proteins (45). Hyperglycemic state, as a key feature in diabetes, is regarded as a principal contributor to thicken CBM in DR, and AGEs and excessive ROS play dominant roles in this thickening process (46). The generation of AGEs on the collagen incurs the cross-linking among collagen proteins, leading to structural stiffness and limiting the transmembrane conveyance of multiple growth factors, and all these pathological alterations finally cause retinal endothelial cell and pericyte death (47). The treatment of aminoguanidine, one AGE inhibitor, plays a protective role to antagonize the thickening of retinal CBM in diabetic rats, which further proves the role of AGEs in this progression (48). The generation of AGEs promotes the accumulation of ROS to indirectly activate the corresponding transcription factors and cytokines that ultimately enhance the levels of ECM proteins in the retinal endothelial cells such as collagen and fibronectin (FN) (45). Fibrosis and thickened CBMs can be attributed to ROS-mediated upregulation of ECM genes (49).

The blood–retinal barrier controls the substance exchange between circulating blood vessels and neural retinal cells. It is regarded as one barrier of high selectivity that can provide essential nutrients and eliminate metabolic waste to maintain the normal function of neural retina. It has been documented that the dysfunction of BRB is closely associated with AGEs (50). AGEs can augment the adhesion of leukocytes that are deeply involved in the dysfunction of the BRB (51). Additionally, AGEs contribute to the breakdown of redox balance and prompt oxidative stress in retinal cells. Dysfunction of the BRB is partly attributed to excessive accumulation of ROS in retinal cells, which induces retinal pericyte loss—a histopathologic hallmark of DR (5).

The formation of acellular and occluded capillaries in DR can be ascribed to the elevated ability of angiogenesis. AGEs can enhance the transcription activities of activator protein-1 and NF-κB to upregulate the expression of angiogenesis-associated genes such as VEGF and angiopoietin-2 (50). In multiple pathological statuses, angiogenesis and inflammation are tightly cohesive processes. AGEs, as the booster for inflammation (52), indirectly prompt the formation of acellular and occluded capillaries. The interaction between AGEs and the RAGE promotes the production of ROS and the activation of NF-κB. The cross talk of ROS and NF-κB signaling is common in retinal cells, and they mutually modulate each other to augment inflammation (5).

### AGEs and neurodegeneration in DR

Retinal neurons, glial cells, and vascular endothelium form a retinal neurovascular unit, a complicated entity of functional and physical coupling, and each component in this entity is closely synchronized to unite retinal blood flow and the metabolic system (53–55). Long-term hyperglycemia facilitates the formation of AGEs and ROS, disrupts the balance of the metabolic system, and then causes the production of inflammatory mediators and cellular damages, which constitute a vicious cycle. A vicious cycle in retinal neurons or vascellum contributes to the dysfunction of this neurovascular unit (56), which can be exemplified by the fact that excessive accumulation of AGEs has been discovered in retinal glial cells, the axons of retinal ganglion cells (RGCs), and the neurons near the retina inner surface (57, 58). The expression of RAGE is also abnormally upregulated in glial cells and RGCs, making these cells susceptible to AGE-involved processes, such as ROS formation and the activation of NF-κB and PKC pathways (57). AGE-induced activation of PKC functions as an upstream positive regulator of NOX, which brings about superabundant NOX-mediated ROS formation and subsequently causes ischemic loss of RGCs, to facilitate neurodegeneration (59). Additionally, early activation of the innate immune and complement systems and microglia also jeopardize a retinal neurovascular unit (60). AGEs can prompt the generation of proinflammatory cytokines and act as persistent antigenic stimulus to be immunostimulatory and impair the retinal neuron (60).

## Therapeutic potential of phytochemicals in DR

Abnormalities associated with AGEs, such as inflammation and oxidative stress, can be used as potential therapeutic targets for DR. Antioxidant phytochemicals are well known for their anti-inflammatory, antioxidant, or anticarcinogenic properties, and their functions in DR treatment have also been investigated by many studies (5, 61, 62). Here, we described some antioxidant phytochemicals and their therapeutic potentials against AGE-mediated abnormality in DR.

Epigallocatechin-3-gallate (EGCG) is the primary polyphenol in green tea, with strong antioxidant capacity. EGCG blocks the formation of AGEs and exerts a curative effect on AGEs-induced collagen cross-linking (63). EGCG can also repress the activity of NF-κB, which plays an antagonistic role in vascular inflammation and apoptosis of retinal cells (64). In addition to the alleviation of oxidative stress, it can also protect the retinal nervous system, and ameliorate the injuries occurring in the BRB and the damage of electroretinograms and basement membrane thickening (64, 65).

Quercetin, a natural flavonoid, can prompt the upregulation of an ROS eraser, e.g., catalase and superoxide-dismutase (SOD), suppress AGE-induced NF-κB activity, and effectively guard against neurodegeneration and ROS-mediated impairments in the retina (66).

Resveratrol, a polyphenol of nonflavonoid phytochemical, is an excellent scavenger to eliminate ROS and exert protective effects against DR (67). It can block ROS-mediated cellular apoptosis in capillary endothelial cells of the retina (68), and also dose-dependently suppress AGE-associated factors, VEGF, TGF-β1, and PKC-β (69).

Curcumin, a polyphenol in *Curcuma longa*, possesses antioxidant, hypoglycemic, and anti-inflammatory capacity and also exhibits therapeutic effects in the treatment of DR (70). It can prevent structural alterations in the retina and block the thickness of CBM in DR (71).

Besides polyphenols, other antioxidants also exert effective effects in DR. Astaxanthin, as a xanthophyll carotenoid, possesses robust antioxidant capacity against oxidative stress. Notably, astaxanthin blocks the formation of endogenous N(ε)-CML, a representative member of AGEs, *via* suppressing ROS. Microalgae extracts containing astaxanthin ameliorate AGEs-induced impairments in retinal pigment epithelial (RPE) cells such as the abnormal expression of VEGF and matrix metalloproteinases (72). A relevant clinical study has revealed that lutein, one phytochemical of the carotenoid family, exerts safe and protective effects on visual function in patients of age-related macular degeneration (73). Lutein ameliorates ischemia-reperfusion injury and represses the apoptosis of retinal pigment epithelial and ganglion cells (74, 75).

Although multiple studies support the therapeutic functions of antioxidant phytochemicals against the progression of DR, the function of one single phytochemical compound is still limited. In patients with diabetes, the multiplex nutritional formula (including resveratrol, the extract of turmeric root and green tea, and other components) can ameliorate visual function and inhibit serum inflammatory factors, which confirms the therapeutic potential of these antioxidants (76). However, some studies show no alleviation of the severity in patients with retinopathy supplied with antioxidants (77). Thus, there is still no explicit conclusion for these antioxidants that has been drawn from the clinical trials. Clinical outcomes are impacted by multiple factors, e.g., the antioxidant dose, the duration of administration, and BRB-influenced transport. Despite these difficulties, it is still encouraging to push the research forward.

## Conclusion

Diabetic retinopathy is a common diabetic complication in patients with diabetes, and the accumulation of AGEs is tightly associated with multiple disorders in the progression of DR, such as metabolic abnormalities, lipid peroxidation, structural and functional alterations, neurodegeneration, and so on. AGEs induce the disruption of the balance between the formation and elimination of ROS and cause oxidative stress in retinal cells, exerting cytopathic effects in this pathophysiology. Antioxidant phytochemicals, as AGE formation inhibitors, are a class of chemicals with reductive and biological activities and possess therapeutic potential in DR treatment, which provide a promising way to control this vision-damaging complication.

## Author contributions

QK conceptualized the topic, researched and analyzed the literature, and wrote the manuscript. HD and SJ constructed the figures. LY revised the manuscript critically. All authors approved the final version of the manuscript, ensure the accuracy and integrity of the work, and agree to be accountable for all aspects of the work.

## Funding

This review is supported by the Chinese National Major Project for New Drug Innovation (2019ZX09201002003), National Natural Science Foundation of China (82030076, 82070161, 81970151, 81670162, and 81870134), Shenzhen Science and Technology Foundation (JCYJ20190808163601776 and JCYJ20200109113810154), Shenzhen Key Laboratory Foundation (ZDSYS20200811143757022), and Sanming Project of Medicine in Shenzhen (SZSM202111004).

## Conflict of interest

The authors declare that the research was conducted in the absence of any commercial or financial relationships that could be construed as a potential conflict of interest.

## Publisher's note

All claims expressed in this article are solely those of the authors and do not necessarily represent those of their affiliated organizations, or those of the publisher, the editors and the reviewers. Any product that may be evaluated in this article, or claim that may be made by its manufacturer, is not guaranteed or endorsed by the publisher.

## Abbreviations

DR: diabetic retinopathy
ROS: reactive oxygen species
Nox: NADPH oxidase
AGEs: advanced glycation end products
DAG: diacylglycerol
VEGF: vascular endothelial growth factor
PKC: protein kinase C
GAPDH: glyceraldehydes-3-phosphate dehydrogenase
RAGEs: receptor for AGEs
GFAT: fructose-6-phosphate amidotransferase
UDP-GlcNAc: diphosphate uracil-N-acetylglucosamine
HHE: hydroxyhexenal
HNE: hydroxynonenal
PUFAs: polyunsaturated fatty acids
DHA: docosahexaenoic acid
AA: arachidonic acid
OA: oleic acid
RGCs: retinal ganglion cells
RPE: retinal pigment epithelium
CBM: capillary basement membrane
ECM: extracellular matrix
NADPH: nicotinamide adenine dinucleotide phosphate
BRB: blood–retinal barrier.
